# 14-3-3η is a novel mediator associated with the pathogenesis of rheumatoid arthritis and joint damage

**DOI:** 10.1186/ar4547

**Published:** 2014-04-21

**Authors:** Walter P Maksymowych, Désirée van der Heijde, Cornelia F Allaart, Robert Landewé, Gilles Boire, Paul P Tak, Yuan Gui, Aziz Ghahary, Ruhangiz Kilani, Anthony Marotta

**Affiliations:** 1 Department of Medicine, University of Alberta, 562 Heritage Medical Research Building, Edmonton, AB T6G 2S2, Canada; 2 Department of Rheumatology, C1R, Leiden University Medical Center, PO Box 9600, 2300 RC Leiden, The Netherlands; 3 Department of Clinical Immunology and Rheumatology, Academic Medical Center, University of Amsterdam & Atrium Medical Center, Meibergdreef 9, Amsterdam 1105AZ, The Netherlands; 4 Service de rhumatologie, Département de médecine, Faculté de médecine et des sciences de la santém, Université de Sherbrooke, 3001-12e Avenue Nord, Sherbrooke, Québec J1H 5N4, Canada; 5 Department of Clinical Immunology and Rheumatology, Academic Medical Center, University of Amsterdam & Atrium Medical Center, Meibergdreef 9, Amsterdam 1105AZ, The Netherlands; 6 GlaxoSmithKline, Stevenage, UK; 7 Augurex Life Sciences Corp, 887 Great Northern Way, Vancouver, BC V5T 4T5, Canada; 8 Department of Surgery, University of British Columbia, 818 West 10th Avenue, Vancouver, BC V5Z 1M9, Canada

## Abstract

**Introduction:**

The aim of this study was to investigate whether 14-3-3η, a specific isoform of a family of proteins regulating processes such as cellular signalling, activates cell-signalling pathways and induces factors known to contribute to the pathophysiology of rheumatoid arthritis (RA). We also investigated whether 14-3-3η is associated with more severe disease in both early and established RA.

**Methods:**

We investigated the effect of 14-3-3η on the activation of RA-relevant signalling cascades and induction of proinflammatory mediators that contribute to the joint damage process. 14-3-3η titres from 33 patients with early RA (mean RA duration = 1.8 months) and from 40 patients with established RA were measured in serum drawn at the 3-year time point of the Behandel Strategieën study. The relationship between 14-3-3η titres and standard clinical variables was investigated by correlation analysis. The association with radiographic damage and radiographic progression over at least a 2-year period was investigated using univariate and multivariate regression analyses.

**Results:**

14-3-3η activated selected members of the mitogen-activated protein kinase (MAPK) family, mainly extracellular regulated kinase 1/2 and c-Jun kinase, but not p38MAPK. Activation by 14-3-3η, using levels spanning the concentration range found in RA patient serum, resulted in the induction of inflammatory transcripts such as interleukin 1 (IL-1) and IL-6 and factors linked to the joint damage process, such as receptor activator of nuclear factor κB ligand and matrix metalloproteinase 1. Serum 14-3-3η correlated significantly with rheumatoid factor (RF) (*r* = 0.43) and anticitrullinated protein antibodies (ACPAs) (*r* = 0.31) in the early RA cohort, but not with C-reactive protein (CRP) or the Disease Activity Score in 28 joints in either cohort. Serum 14-3-3η concentrations were significantly higher in patients with radiographically assessed joint damage and in those who had radiographic progression. By multivariate analysis, we show that 14-3-3η complemented markers such as CRP, RF and ACPA in informing RA radiographic status and/or progression.

**Conclusions:**

Extracellular 14-3-3η activates key signalling cascades and induces factors associated with the pathogenesis of RA at concentrations found in patients with RA, and its expression is higher in patients with radiographic damage and RA progression.

## Introduction

Rheumatoid arthritis (RA) is a chronic autoimmune disease characterised by inflammation of the synovium [1]. When left untreated, RA typically leads to severe joint destruction [2]. Early diagnosis, combined with an accurate prognostic assessment at presentation, is a central tenet in the effective management of RA patients [3]. There is consensus amongst rheumatologists that the development of a risk stratification strategy to group patients into low-, medium- and high-risk categories for radiographic progression at presentation is a clinical imperative [4]. Currently, this remains a major limitation in patient management because risk factors such as rheumatoid factor (RF), anticitrullinated protein antibodies (ACPAs; often detected by performing an anti–cyclic citrullinated peptide antibody test) and C-reactive protein (CRP) together account for only 32% of the total variance in predicting joint destruction, leaving 68% of the variance unaccounted for [4].

Serum 14-3-3η, which was first described in 2007 [5] as being elevated in arthritis, has been reported to be significantly higher in RA patients than in healthy individuals and in various disease controls including, but not limited to, osteoarthritis (OA), ankylosing spondylitis (AS) and gout [6,7]. On the basis of receiver operating characteristic curve analyses defining optimal sensitivity and specificity, serum 14-3-3η positivity is denoted by a concentration ≥0.19 ng/ml [6,7]. We have previously shown that (1) serum 14-3-3η expression is not strongly correlated with standard clinical and serological measures, is detected in both early and established RA, with sensitivities of 60% to 82% and 78%, respectively, and (2) that 14-3-3η positivity adds incrementally to both RF and ACPA positivity for diagnostic sensitivity [8]. In a previous study of an early RA cohort, we reported that 60% of patients were positive for 14-3-3η, 32% for RF, 44% for ACPA and 72% for at least one of those three markers [8].

The 14-3-3 family of conserved regulatory proteins consists of seven isoforms: α/β, γ, δ/ζ, ε, η, θ/τ and σ. Under normal circumstances, these proteins exist as intracellular adapters that can either homo- or heterodimerise to form a cuplike structure known as the *amphipathic groove*, which allows them to interact with more than 200 intracellular proteins to modulate their activities. Interactions include an array of biological processes, such as protein trafficking and cellular signalling. Externalisation of 14-3-3η, similarly to its process in RA [5], is believed to be mediated in part through an exosomal process in a like that of other intracellular proteins, such as the release of heat shock proteins (HSPs) from human peripheral blood mononuclear cells (PBMCs) or B lymphocytes under heat stress [9,10]. Importantly, these two families of proteins—14-3-3 and HSPs—have been described as key components of exosomal microvesicles [11], and it is now widely accepted that members of the HSP superfamily, such as HSP60, behave as proinflammatory factors priming the immune system to respond to a noxious agent [12].

In 2007, we reported a strong correlation between the expression of 14-3-3η and matrix metalloproteinases (MMPs) and demonstrated that extracellular 14-3-3η possesses MMP-1-inducing activity *in vitro* on the basis of 14-3-3η levels at the upper range of serum levels observed in a small subset of RA patients [5-7]. MMPs are serine proteases that play a critical role in maintaining tissue homeostasis, but, in the context of RA, the imbalance between expression of these proteolytic enzymes and their cognate inhibitors leads to the breakdown of cartilage [13].

MMP expression has been reported to be regulated through the transcription factor activator protein 1 (AP-1), which resides downstream from intracellular signalling factors such as mitogen-activated protein kinase (MAPK) [14-17]. The MAPK family, which includes extracellular regulated kinase (ERK), c-Jun N-terminal kinase/stress-activated protein kinase (JNK/SAPK) and p38MAPK, has been investigated extensively in RA [18]. Recently, de Launay *et al*. reported a specific increase in ERK and JNK activation, but not in p38MAPK activation, in early RA patients with progressive joint destruction, underscoring their possible relevance to the aetiology of RA [19].

In our present study, given the strong correlation of 14-3-3η with MMP-1 and MMP-3 in synovial fluid and serum [5], we sought to advance the understanding of the role of 14-3-3η in RA by assessing whether 14-3-3η (1) possesses extracellular ligand properties leading to activation of MAPK, (2) induces mediators of inflammation and joint destruction and (3) contributes prognostic information in addition to clinical and serological measures used in the management of RA.

## Methods

### 14-3-3η effects on cell signalling and induction of pro-inflammatory mediators

To examine whether 14-3-3η possesses extracellular ligand properties relevant to RA, we investigated its effects on the activation of RA-relevant signalling cascades and the induction of proinflammatory mediators that contribute to the joint damage process.

Low-endotoxin human recombinant 14-3-3η (Augurex Life Sciences Corp, North Vancouver, BC, Canada) and human recombinant tumour necrosis factor α (TNFα) from (R&D Systems, Minneapolis, MN, USA) were used for cell stimulation. 14-3-3η and TNFα were diluted using serum-free media whereby 5 μl of the 14-3-3η or TNFα stock were added to each millilitre of medium in wells to achieve the concentration used for cell stimulation dosing. A dose selection study (0 to 100 ng/ml), coupled with a 15-minute cell stimulation experiment, was performed to select the doses and time points for assessment of cell signalling pathways, which were analysed by immunoblotting with a phosphotyrosine antibody. On the basis of the results of this study, we determined that, for cell signalling experiments, a minimum dose of 12.5 ng/ml 14-3-3η and TNFα would be used in order to observe a visibly detectable difference in phosphorylation status above the unstimulated control. This dose of extracellular 14-3-3η reflects concentrations observed in patient sera (0.0 to >20.0 ng/ml). Kilani *et al.*[5] reported that 14-3-3η expression detected by Western blot analysis was three to five times higher in synovial fluid than in matched patient serum, supporting the 12.5 ng/ml cell stimulation dose as being consistent with the concentration of 14-3-3η present in the joint. For transcriptional studies of proinflammatory mediators, we analysed the impact of a range of 14-3-3η concentrations comparable to those detected in RA patient serum (0.1 to 100 ng/ml) and found that they were well within the range found in synovial fluid.

### Cell culture

Monocyte lineage THP-1 cells were selected to investigate the stimulatory effects of 14-3-3η, as this cell line has been used extensively in the field because of the importance of this lineage of cells in the pathogenesis of RA [20]. Briefly, THP-1 cells were cultured in RPMI 1640 medium supplemented with 10% foetal bovine serum in a humidified chamber at 37°C in a 5% CO_2_ atmosphere. Prior to being seeded, cells were centrifuged for 5 minutes at 2,000 rpm. The cell pellet was then washed once gently with serum-free medium, centrifuged and resuspended to 1 × 10^6^ cells/ml.

### Activation of cell signalling pathways

To determine which cell signalling pathways are specifically activated by 14-3-3η, the THP-1 cells were seeded at a density of 2 × 10^6^ cells per well and deprived of serum for 6 hours prior to cell stimulation. Following cell starvation, the cells were stimulated with 12.5 ng/ml of ligand (14-3-3η or TNFα) up to 30 minutes. Cells were harvested and lysed, and the phosphorylation status of MAPK family members, specifically ERK, JNK/SAPK and p38MAPK, were evaluated using phosphorylated MAPK and the corresponding pan-antibodies (Cell Signaling Technology, Beverly, MA, USA). Changes in phosphorylation status were measured by densitometry using ImageJ software (National Institutes of Health, Bethesda, MD, USA). Total protein levels were used as a background control to ensure that changes were due to increases in phosphorylation status rather than to increases in the respective kinases.

### Expression of proinflammatory mediators

Induction of mediators of inflammation and destruction by a range of concentrations of 14-3-3η (0.1 to 100 ng/ml) were evaluated by mRNA analysis for interleukin 1β (IL-1β), IL-6, MMP-1, MMP-9, receptor activator of nuclear factor κB ligand (RANKL) and TNFα. THP-1 cells were resuspended in medium containing 0.1% foetal bovine serum, seeded at a density of 2 × 10^6^ cells per well into a six-well dish and allowed to stand for 2 hours. The cells were then stimulated with 14-3-3η (0.1 to 100 ng/ml) or TNFα (50 ng/ml) for 18 hours using 5 μl of ligand per millilitre of culture medium added to each well. The reaction was terminated by the addition of ice-cold phosphate-buffered saline, and then the cells were centrifuged. Following centrifugation, RNA was extracted from the cell pellets using the illustra RNAspin Mini RNA Isolation Kit (GE Healthcare Life Sciences, Pittsburgh, PA, USA). The cDNA was synthesised from purified total RNA using the RevertAid™ H Minus First Strand cDNA Synthesis Kit (Thermo Scientific, Pittsburgh, PA, USA). The PCR conditions for each primer were optimised independently. Primers for RT-PCR analyses were produced either by Augurex Life Sciences Corp or purchased from R&D Systems. The levels of the housekeeping gene *GAPDH* were evaluated to ensure that equal amounts of cDNA were used for all samples.

### Rheumatoid arthritis cohorts

#### Cohort A

Cohort A comprised 33 patients with RA (defined according to the 1987 American Rheumatism classification criteria [19]) who were members of the Early Undifferentiated PolyArthritis (EUPA) Cohort of the University of Sherbrooke. Adult patients with synovitis affecting at least three joints for 1 to 12 months were followed longitudinally as previously described [21]. The mean age (SD) of the patients was 51.1 years (5.0), 23 (70%) of the patients were female, the median (IQR) disease duration was 1.8 months (1.5 to 2.8) and the median (IQR) Disease Activity Score in 28 joints (DAS28) was 5.9 (4.8 to 6.5). Serological assessment comprised erythrocyte sedimentation rate (ESR), CRP, RF and ACPA, and radiographic assessments were performed using the Sharp/van der Heijde Score (SHS). Radiographs obtained at baseline and at the 30-month follow-up examination were available. Radiographic progression was determined based on the change in SHS at 30 months. All radiographs were read in known time sequence by one or two trained reviewers blinded to patient characteristics and treatment. At baseline, all patients were disease-modifying anti-rheumatic drug (DMARD)–naïve. Patients were treated with the objective of rapidly attaining a zero swollen joint count. During the 30-month follow-up period, 29 patients (88%) received conventional DMARD therapy and 5 (15%) received oral steroid therapy, and 4 patients were receiving anti-TNF therapy at the time of the 30-month evaluation.

#### Cohort B

Cohort B comprised 40 patients with established RA at the year 3 time point of the Behandel Strategieën (BeSt) study [22]. The patients’ mean age (SD) was 50.9 years (12.2), and 30 (75%) of the patients were female. The median (IQR) DAS28 score was 1.9 (1.5 to 2.6). In the BeSt study, patients were assigned to one of four treatment strategies: sequential DMARD monotherapy (group 1), step-up combination therapy (group 2), initial combination therapy with tapered high-dose prednisone (group 3) and initial combination therapy with the TNF inhibitor infliximab (group 4). The patients in our present 14-3-3η study were selected from among groups 1 through 3. Patients in group 4 were not included, because, as we have previously reported, 14-3-3η is a TNF-responsive gene whose levels are modifiable with TNF inhibitor therapy in both RA and psoriatic arthritis patients [6,23]. Treatment may therefore have a confounding effect on the assessment of 14-3-3η’s association with radiographic progression, even in groups 1, 2 and 3.

Serological measurements for CRP, RF and ACPA were analysed, and joint assessments performed, using the SHS as previously described [22,24]. Radiographs were available for the 3- and 5-year time points of our present study. Radiographic progression was determined based on the change in the SHS at year 5.

This study was performed in accordance with the Declaration of Helsinki, and the study protocol was approved by the Health Research Ethics Review Board of the University of Sherbrooke and the Ethics Review Board of Leiden University. All patients provided their written informed consent to participate in the study.

### Radiographic assessment

To investigate 14-3-3η’s clinical association with radiographic joint damage and joint damage progression for both the early and established RA cohorts, we compared the groups according to the presence or absence of joint damage at the start of the study (SHS <1 or ≥1) and according to the presence or absence of radiographic progression at the end of the follow-up period (ΔSHS <1 or ≥1).

### Serum 14-3-3η measurements

Serum 14-3-3η levels were measured using a 14-3-3η enzyme-linked immunosorbent assay (Augurex Life Sciences Corp). Serum samples were diluted 1:20 using the supplied assay dilution buffer. A 100-μl volume of either the corresponding standard or sample was incubated in a 96-well plate shaker for 2 hours at 500 rpm and 27°C. Following incubation, the plates were washed four consecutive times with wash buffer and then incubated with the prediluted horseradish peroxidase–conjugated antibody for 1 hour at room temperature without shaking. The plates were washed four additional times and subsequently incubated with 3,3′,5,5′-tetramethylbenzidine substrate for 30 minutes, at which time the reaction was terminated by the addition of 1 N HCl stop solution. Serum 14-3-3η levels were quantified using a four-parameter logistic regression curve generated against the diluted standards. The accuracy of the results were evaluated by examining the precision of the back-calculated concentrations of each of the standards and the corresponding measurements of three quality control samples with known 14-3-3η levels within the linear range of the assay. All clinical samples were run in duplicate. Samples with levels below the reportable range were assigned a concentration of 0.0 ng/ml, and those with levels above the upper limit were defined as having levels >20 ng/ml. Actual 14-3-3η concentrations for samples with values >20 ng/ml were determined by serial dilution and used for statistical analysis.

### Statistical analysis of clinical 14-3-3η expression

The Mann–Whitney *U* test was used to compare median differences between groups. A two-tailed Student’s *t*-test with Welch’s correction was employed to account for significant variances between groups. Pearson product–moment correlations were calculated to examine the relationship between 14-3-3η and clinical and serological measurements, including DAS28 and titres of CRP, RF and ACPA.

Fisher’s exact test was used to compare the association between 14-3-3η positivity, at various positivity cut points, of RF or ACPA with radiographic damage status (SHS <1 or ≥1) and radiographic progression (ΔSHS <1 or ≥1). 14-3-3η positivity cutoffs were defined at the diagnostic positivity cutoff of ≥0.19 ng/ml, approximately twice the diagnostic cutoff (0.4 ng/ml) and four times the diagnostic cutoff (0.8 ng/ml). Univariate regression analyses were performed to identify which clinical and serological markers were associated with joint damage status and radiographic progression in the two cohorts. Further regression analyses were performed to identify variables associated with radiographic damage status and/or progression by combining only those variables that had a *P*-value ≤0.10 in univariate analysis. The strength of the association is expressed using χ^2^ likelihood ratios with 95% confidence intervals (CI), together with the total variance, to inform the primary outcome (*R*^2^).

All statistical analyses were performed using Prism 6 software (GraphPad Software, La Jolla, CA, USA) and JMP9 software (SAS Institute, Cary, NC, USA). Results were considered significantly different at *P* < 0.05.

## Results

### A pathophysiologic role for extracellular 14-3-3η

**Figure 1:**
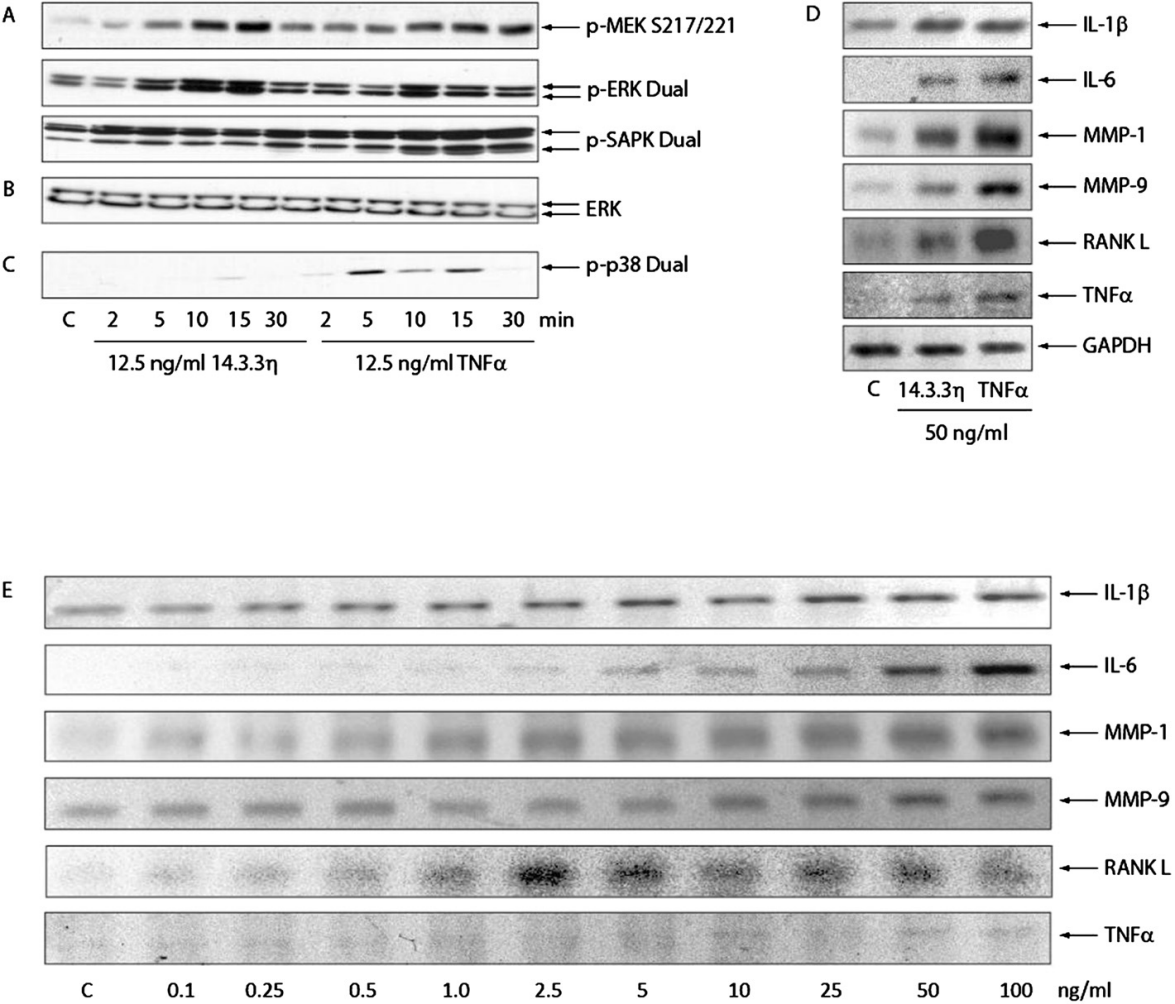
**Extracellular 14-3-3η selectively activates extracellular signal-regulated kinase and c-Jun N-terminal kinase/stress-activated protein kinase and potentiates inflammatory and joint damage factors. (A)** 14-3-3η leads to the phosphorylation and activation of extracellular signal-regulated kinase (ERK) and c-Jun N-terminal kinase (JNK). p-MEK, Phosphorylated mitogen-activated protein kinase kinase; p-SAPK, Phosphorylated stress-activated protein kinase. **(B)** Equal protein loading. **(C)** The effects were specific for ERK and JNK because phosphorylation of p38 mitogen-activated protein kinase (p38MAPK) by 14-3-3η was not observed. **(D)** Similarly to tumour necrosis factor α (TNFα), 14-3-3η leads to the induction of inflammatory and joint damage factors. IL, Interleukin; GAPDH, Glyceraldehyde 3-phosphate dehydrogenase; MMP, Matrix metalloproteinase; RANKL, Receptor activator of nuclear factor κB ligand. **(E)** 14-3-3η induced effects observed with 14-3-3η concentrations that closely approximated the 14-3-3η median rheumatoid arthritis (RA) serum concentrations.

Stimulation of THP-1 cells with recombinant 14-3-3η resulted in an increase in the phosphorylation of ERK and JNK/SAPK by approximately 87% and 227% above control at 2 and 5 minutes, respectively (Figure 1A to 1C). In contrast to TNFα, no phosphorylation of p38MAPK by 14-3-3η was observed at any time point evaluated. These results suggest that 14-3-3η and TNFα possess both common and unique signalling effects.

As depicted in Figure 1D and 1E, stimulation of THP-1 cells with 14-3-3η resulted in the dose-dependent induction of inflammatory transcripts and factors directly linked to the joint damage process, such as IL-1β, IL-6, MMP-1, MMP-9 and RANKL. Similar effects were observed with TNFα when the same concentration was used. Induction of transcripts by 14-3-3η was evident with concentrations as little as 1.0 ng/ml across all of the factors tested (Figure 1E), which corresponds with levels reported in patients with RA (Table 1). Certain transcripts, such as IL-1β and MMP-1, were determined to be more sensitive to stimulation by 14-3-3η, which was evident even as low as 0.1 ng/ml.

**Table 1:** **Patient characteristics based on radiographic progression in two cohorts of rheumatoid arthritis patients**^**a**^

	**Early RA (*n*** **= 33)**			**Established RA (*n*** **= 40)**		
	**No progression (SHS <1)**	**Progression (SHS ≥1)**	***P*-value (U-test)**	**No progression (SHS <1)**	**Progression (SHS** ≥**1)**	***P*-value (U-test)**
Patients, *n*	13	20	–	20	20	–
DAS28 ESR	5.0 (4.4 to 5.7)	6.1 (5.3 to 6.5)	0.04	1.9 (1.4 to 2.3)	1.9 (1.6 to 2.7)	0.56
Mean age (SD), yr	50.9 (5.2)	51.3 (5.0)	0.82	50.3 (10.6)	51.5 (13.9)	0.77
Sex, *n* (% female)	10 (77%)	13 (65%)	–	14 (70%)	16 (80%)	–
Baseline SHS	0 (0 to 0)	2.5 (1.0 to 6.8)	0.0002	1.0 (0 to 16)	38.7 (23.2 to 56.8)	<0.0001
CRP, mg/L	12.0 (6.0 to 22.5)	12.5 (6.5 to 24.5)	0.82	3.0 (2.0 to 6.0)	8.0 (5.3 to 22.0)	0.001
RF, IU/ml	0 (0 to 160.0)	160.0 (40.0 to 320.0)	0.035	48.0 (2.0 to 68.0)	55.0 (19.5 to 223.8)	0.13
ACPA, U/ml	12 (6.5 to 96.0)	101.0 (14.3 to 194.3)	0.18	59.5 (20.3 to 365.9)	75.1 (20.3 to 440.3)	0.77
Mean 14-3-3η (SD), ng/ml	1.30 (3.51)	6.13 (8.33)	0.03	3.76 (6.92)	4.37 (8.02)	0.79
Median 14-3-3η (IQR), ng/ml	0.09 (0.06 to 12.59)	2.68 (0.12 to 15.94)	0.006	0.28 (0 to 4.54)	1.11 (0.10 to 2.89)	0.16

^a^ACPA, Anticitrullinated protein antibody; SHS, Sharp/van der Heijde score; CRP, C-reactive protein; DAS28 ESR, Disease Activity Score in 28 joints with erythrocyte sedimentation rate; RA, Rheumatoid arthritis; RF, Rheumatoid factor. P-values were calculated using the Mann–Whitney *U* test.

### Relationship of 14-3-3η with clinical and serological measures

Table 2 provides the correlations between the clinical and serological variables. In both the early and established RA cohorts, 14-3-3η titres did not correlate significantly with DAS28 scores or CRP levels. There was a modest correlation with RF (*r* = 0.43, *P* < 0.01) and ACPA (*r* = 0.31, *P* < 0.05) in the early RA cohort, but not in the established RA cohort.

**Table 2:** **Correlation of 14-3-3η with clinical and serological measures in two cohorts of patients with rheumatoid arthritis**^**a**^

**Cohort**	**Variable, score or titres**	**DAS28**	**CRP**	**RF**	**ACPA**
A	DAS28				Pearson product–moment correlation (*r*)
	CRP	0.12			
	RF	0.22	-0.13		
	ACPA	-0.14	0.20	0.27	
	14-3-3η	0.18	0.20	0.43^b^	0.31^c^
B					
	DAS28				Pearson product–moment correlation (*r*)
	CRP	0.46^d^			
	RF	-0.06	-0.02		
	ACPA	-0.17	-0.14	0.17	
	14-3-3η	-0.03	-0.09	0.03	-0.07

^a^ACPA, Anticitrullinated protein antibody; CRP, C-reactive protein DAS28: Disease Activity Score in 28 joints; RF: rheumatoid factor. ^b^*P* < 0.01; ^c^*P* ≤ 0.05; ^d^*P* < 0.001. Cohort A is the University of Sherbrooke early rheumatoid arthritis cohort at the baseline time point (*n* = 33). Cohort B is the Behandel Strategieën (BeSt) study cohort of patients with established RA at the year 3 time point (*n* = 40).

### 14-3-3η serum concentration and its association with radiographic damage and progression in rheumatoid arthritis

**Figure 2:**
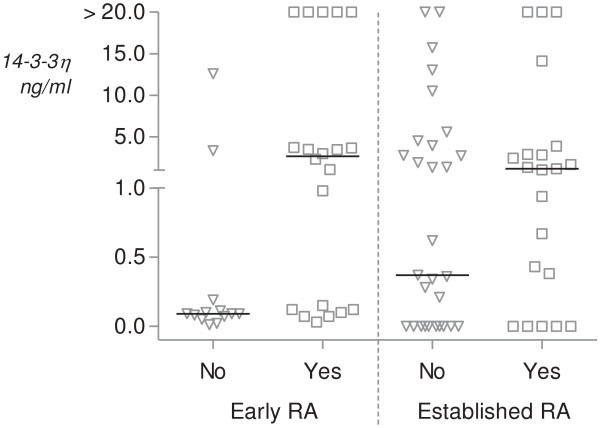
**Scatterplots illustrating serum 14-3-3η concentration according to radiographic progression of rheumatoid arthritis.** Radiographic progression of rheumatoid arthritis (RA) was defined as any change in Sharp/van der Heijde score <1 or ≥1 at the end of the follow-up period (30 months for early RA (*n* = 33) and 2 years for established RA (*n* = 40)). Triangle = patients with no erosion; Square = patients with erosions; Line = mean levels.

Twenty (61%) of the thirty-three early RA patients with 30-month follow-up data developed radiographic progression and twenty (50%) of forty RA patients in the BeSt cohort had radiographic progression. The characteristics of the patients in the two cohorts, who were grouped according to radiographic progression (ΔSHS <1 or ≥1), are provided in Table 1. Patients in both groups were well-matched for age and sex. In the early RA patient group, the median baseline DAS28 score was higher in the progression group than in the no-progression group (6.1 vs. 5.0; *P* = 0.04), whereas baseline DAS28 scores in the established RA cohort did not differ significantly between the progression and no-progression groups (1.9 vs. 1.9; *P* = 0.56). Median (IQR) baseline 14-3-3η levels were significantly higher in early RA patients with radiographic progression compared with those whose RA did not progress (2.68 ng/ml (0.12 to 15.94) vs. 0.09 ng/ml (0.06 to 12.59); *P* = 0.006). 14-3-3η levels were also higher in established RA patients with radiographic progression. However, these levels were not significantly different (1.11 ng/ml (0.10 to 2.89) vs. 0.28 ng/ml (0 to 4.5); *P* = 0.16). The respective scatterplots for 14-3-3η serum concentrations according to radiographic progression (ΔSHS <1 or ≥1) are provided in Figure 2.

Univariate analysis revealed that the strength of the association of radiographic damage at baseline and radiographic damage progression increased with higher 14-3-3η positivity cut points in both the early and established RA cohorts (Table 3). Using the diagnostic cutoff of ≥0.19 ng/ml, the odds ratio (OR) of radiographic damage progression in early RA was 6.2 (95% CI = 1.3 to 30.2; *P* = 0.022), which increased to 10.2 (95% CI = 1.7 to 59.7; *P* = 0.006) with cutoffs of ≥0.40 ng/ml and ≥0.80 ng/ml, respectively. RF positivity was also determined to be associated with radiographic progression in the early RA cohort, with an OR of 9.1 (95% CI = 1.7 to 47.7; *P* = 0.008). In the established RA cohort, the OR of radiographic progression was similarly higher at the positivity cutoff of ≥0.40 ng/ml (OR = 4.3 (95% CI = 1.2 to 16.3; *P* = 0.028) than at the cutoff of ≥0.19 ng/ml (OR = 2.5 (95% CI = 0.6 to 9.4; *P* = 0.18). The CRP titre was the only other variable determined to be associated with radiographic progression in the established RA cohort (OR = 1.1 (95% CI = 1.0 to 1.4; *P* = 0.003).

**Table 3:** **Univariate association of clinical and laboratory variables with damage status and radiographic outcomes**^**a**^

**Variable**	**Early RA (*n*** **= 33)**				**Established RA (*n*** **= 40)**			
	**Baseline damage status**		**Progression**		**Baseline damage status**		**Progression**	
	**OR (95% CI)**	***P*-value**	**OR (95% CI)**	***P*-value**	**OR (95% CI)**	***P*-value**	**OR (95% CI)**	***P*-value**
DAS28 ESR	1.2 (0.7 to 2.3)	0.485	**1.7 (0.9 to 3.6)**	**0.094**	1.5 (0.5 to 4.9)	0.496	1.4 (0.6 to 3.5)	0.475
Age	1.0 (0.8 to 1.1)	0.505	1.0 (0.9 to 1.2)	0.815	1.1 (1.0 to 1.2)	0.112	1.0 (1.0 to 1.1)	0.763
Sex	2.8 (0.6 to 13.8)	0.192	1.8 (0.4 to 8.7)	0.462	1.0 (0.2 to 6.0)	0.660	1.7 (0.4 to 7.3)	0.358
CRP	**1.0 (1.0 to 1.1)**	**0.084**	1.0 (1.0 to 1.0)	0.906	**1.6 (1.1 to 3.1)**	**0.004**	**1.1 (1.0 to 1.4)**	**0.003**
ACPA + ve	1.1 (0.3 to 4.4)	0.580	2.2 (0.5 to 9.0)	0.239	**7.7 (1.3 to 45.3)**	**0.022**	2.2 (0.6 to 8.5)	0.214
RF + ve	2.5 (0.6 to 11.3)	0.195	**9.1 (1.7 to 47.7)**	**0.008**	**10.3 (1.7 to 62.7)**	**0.010**	1.2 (0.3 to 4.5)	0.500
14-3-3η								
+ve ≥0.19 ng/ml	**4.0 (0.9 to 17.2)**	**0.057**	**6.2 (1.3 to 30.2)**	**0.022**	**9.0 (1.5 to 53.9)**	**0.014**	2.5 (0.6 to 9.4)	0.160
+ve ≥0.40 ng/ml	**5.5 (1.2 to 24.8)**	**0.025**	**10.2 (1.7 to 59.7)**	**0.006**	**4.4 (0.8 to 25.2)**	**0.089**	**4.3 (1.2 to 16.3)**	**0.028**
+ve ≥0.80 ng/ml	**5.5 (1.2 to 24.8)**	**0.025**	**10.2 (1.7 to 59.7)**	**0.006**	**7.9 (0.8 to 25.2)**	**0.044**	**3.5 (0.9 to 13.0)**	**0.055**

^a^ACPA, Anticitrullinated protein antibody; CI, Confidence interval; CRP, C-reactive protein; DAS28, Disease Activity Score in 28 joints; ESR, Erythrocyte sedimentation rate; OR, Odds ratio; RA, Rheumatoid arthritis; RF, Rheumatoid factor; +ve, Dependent variable increase for increase in independent variable. Variables with corresponding *P*-value ≤0.10 are presented in bold.

The models presented in Table 4 demonstrate that 14-3-3η positivity adds incrementally to other significant clinical and serological measures in univariate analysis with regard to informing radiographic status and progression. In the early RA cohort, when 14-3-3η positivity (cutoff set at 0.19 ng/ml) is combined with CRP to inform radiographic status, the contribution to the total variance of the outcome (*R*^2^) increases from 0.065 to 0.154. In established RA, when combined with CRP titres, together with RF and ACPA positivity, the contribution to total variance (*R*^2^) increases from 0.376 to 0.425. With respect to radiographic RA progression, when 14-3-3η positivity (cutoff set at ≥0.40 ng/ml) is combined with DAS28 score and RF positivity, the contribution to the total variance in the early RA cohort increases from 0.252 to 0.310. In the established RA cohort, when 14-3-3η positivity is combined with CRP titres, the contribution to the total variance increases from 0.167 to 0.262.

**Table 4:** **Multivariate analyses indicating contribution of 14-3-3η to total variance in radiographic damage status and radiographic progression**^**a**^

**Cohort**	**Outcome**		**Without 14-3-3η**	**With 14-3-3η**		
				**≥0.2 ng/ml**	**≥0.4 ng/ml**	**≥0.8 ng/ml**
Early RA	Joint damage status	Model LR	2.99	7.06	8.54	8.54
		p	0.084	0.029	0.014	0.014
		*R*^2^	0.065	0.154	0.187	0.187
	Radiographic progression	Model LR	11.16	12.27	13.72	13.72
		P	0.004	0.007	0.003	0.003
		*R*^2^	0.252	0.273	0.310	0.310
Established RA	Joint damage status	Model LR	12.35	13.94	12.36	12.76
		P	0.006	0.008	0.015	0.013
		*R*^2^	0.376	0.425	0.377	0.389
	Radiographic progression	Model LR	8.78	9.71	13.78	13.13
		P	0.003	0.008	0.001	0.001
		*R*^2^	0.167	0.185	0.262	0.250

^a^LR, Likelihood ratio; RA, Rheumatoid arthritis.

## Discussion

14-3-3η is one of seven members of the 14-3-3 family that are preferentially expressed at higher concentrations in certain tissues, underscoring the importance of specific isoforms in the regulation of tissue-specific functions [25-27]. Data that we reported in 2007 indicated that 14-3-3η is expressed at significantly higher levels than the other isoforms in the synovial fluid of patients with arthritis and that these levels were three to five times higher than corresponding levels in the serum of matched donors, citing the joint as the likely source of serum 14-3-3η [5]. Since that first report, we have demonstrated that 14-3-3η is an RA-specific marker that complements both RF and ACPA, increasing their diagnostic value [6-8]. We also reported a positive association between 14-3-3η and MMPs and suggested that 14-3-3η may have a role in the pathogenesis of RA [28].

To expand upon our understanding of the biological relevance of extracellular 14-3-3η, we performed *in vitro* cell signalling studies to determine if 14-3-3η signals through the MAPK family, as well as through which family members. This family was selected because the transcription factor AP-1, which resides downstream in the MAPK signalling nexus, is a key regulator of MMP expression. The data produced in this study indicate that stimulation of cells with 14-3-3η, similarly to TNFα, results in the phosphorylation of both ERK and JNK/SAPK in a time-dependent manner. However, unlike TNFα, 14-3-3η had no impact on p38MAPK phosphorylation. The differential effects of 14-3-3η in relation to TNFα on the activation of MAPK family members indicates that these two ligands, which are present in the serum of patients with RA, are likely to signal by divergent mechanisms. Phosphorylation of these two MAPK members, ERK and JNK/SAPK, together with the finding that 14-3-3η is present in the serum of 60% to 82% of early RA patients [8], is consistent with the observations reported by de Launay *et al*.: that ERK and JNK, but not p38MAPK, are preferentially phosphorylated in early RA [19]. On the basis of these findings, we propose that the relationship between 14-3-3η expression and the phosphorylation status of the MAPK family members, especially in early RA, be investigated in human synovial tissue.

It is well-established that phosphorylation of both ERK and JNK/SAPK at the TXY motif within the activation loop of these kinases is reflective of their activation. In turn, these MAPKs directly and/or indirectly phosphorylate the transcription factor c-Jun. Phosphorylation of c-Jun results in its association with c-fos, the formation of the AP-1 transcription complex and the transactivation or switching-on of genes containing the AP-1 binding site [29]. In this head-to-head analysis, we demonstrate that 14-3-3η at a concentration similar to that of TNFα, is capable of inducing genes that have been described in the literature as being TNFα-responsive. Our 14-3-3η dose escalation studies demonstrate that these TNFα-responsive genes are highly sensitive to 14-3-3η stimulation and can be induced with as little as 1.0 ng/ml, a 14-3-3η concentration that approximates median concentrations measured in the serum of RA patients [6-8]. On the basis of these *in vitro* findings, it is evident that 14-3-3η may play a role in perpetuating inflammation through the induction of factors such as IL-6 and by exacerbating joint destruction via MMPs and RANKL. Examining 14-3-3η’s expression in relation to clinical outcomes in RA patients will be of utmost importance in understanding how 14-3-3η serum expression used as a diagnostic test can assist clinicians with patient management, to further substantiate its relevance to the pathophysiology of RA and to gain a better understanding of how 14-3-3η biomarker expression can be aligned with the advancement of a 14-3-3η-targeted therapy to identify the subset of patients who are more likely to respond to such an approach.

High levels of other 14-3-3 isoforms have been associated with more severe disease or worse outcomes in many patient groups, including, but not limited to, carcinoma [30-33], Creutzfeldt-Jakob disease [34], Alzheimer’s disease [35], multiple sclerosis [36], infarction [37] and HIV [38]. Analysis of serum 14-3-3η expression in relation to joint damage and progression revealed significantly higher levels of 14-3-3η in patients who already had radiographic evidence of damage at study baseline, as well as in those who developed progression by the end of the follow-up period. Amongst other laboratory variables tested, none were significantly associated with radiographic status at baseline in the early RA cohort and only RF was significantly associated with radiographic progression. Nevertheless, 14-3-3η contributed to the variance of a regression model for radiographic progression (*R*^2^) that included RF. The association of 14-3-3η with radiographic status and progression was less prominent in the BeSt cohort than in the early RA cohort, but DAS28 control was already very well-established by year 3 in the majority of patients in the BeSt cohort (mean DAS28 = 1.9). Of the three laboratory variables examined—CRP, RF, and ACPA—only CRP was associated with radiographic progression. 14-3-3η contributed to the variance of a regression model for radiographic progression that included CRP.

In this study, we observed that serum 14-3-3η did not correlate with disease activity (DAS28) or with titres of CRP. We noted a modest correlation with ACPA and RF in the early RA cohort, but not in the established RA cohort. These findings indicate that 14-3-3η may act independently of these measures, which is one of the key requirements proposed by the Outcome Measures in Rheumatology (OMERACT) soluble biomarker working group for a biomarker reflecting joint damage [39]. The OMERACT guidelines state that, in order to be useful at informing structural damage endpoints, a marker should be independent of other commonly available variables associated with radiographic progression and should complement them in predictive models. Regression analyses revealed that 14-3-3η added incrementally to the predictive power of the model. On the basis of these findings, a prospective study designed to examine 14-3-3η’s capacity as an independent predictor of radiographic damage status and progression is underway.

As with proof-of-concept studies, one of the main limitations of the present study is the small sample size for both RA cohorts. In addition, relatively few patients demonstrated substantial joint damage progression. The results of this study are being used in the design of larger studies to formally investigate the merits of 14-3-3η in marking joint damage and predicting radiographic progression in combination with other clinical variables. In addition, we have previously shown that 14-3-3η is modifiable by both anti-TNF and standard DMARD therapies [40,41]. Confounding by treatment is a significant limitation of studies in which the predictive role of biomarkers is assessed when only baseline biosamples are available. It is to be anticipated that levels of 14-3-3η are likely to change in both cohorts when treatment changes are driven by DAS28 targets, which will limit the predictive capacity of biosample data that are available only at baseline. This was particularly noteworthy in a previous report of BeSt study patients in which baseline levels of acute-phase reactants, RF and ACPA were not shown to be predictive of radiographic progression in the two patient groups undergoing intensive therapy. However, RF and ACPA were predictive in patients being treated with monotherapy or combination therapy with standard DMARDs [24]. Consequently, future studies should assess biosamples at multiple time points.

## Conclusions

In our present study, we demonstrate that extracellular 14-3-3η is an activator of ERK and JNK, but not p38MAPK. These effects are comparable to, yet distinct from, TNFα. At concentrations present in the serum of RA patients, stimulation of monocytes with soluble 14-3-3η results in the induction of genes that perpetuate inflammation and drive joint damage. Our clinical biomarker results reveal that 14-3-3η expression is higher in the serum of patients with radiographic evidence of damage and progression. The alignment between the *in vitro* findings and the clinical biomarker expression profile provide a strong rationale for investigating 14-3-3η as both a marker of radiographic outcome and possibly a new therapeutic target.

## Abbreviations

ACPA: Anticitrullinated protein antibody; AS: Ankylosing spondylitis; CRP: C-reactive protein; DAS28: Disease Activity Score in 28 joints; DMARD: Disease-modifying antirheumatic drug; ERK: Extracellular signal-regulated kinase; HSP: Heat shock protein; JNK/SAPK: c-Jun N-terminal kinase/stress-activated protein kinase; LR: Likelihood ratio; MAPK: Mitogen-activated protein kinase; MMP: Matrix metalloproteinase; OA: Osteoarthritis; OR: Odds ratio; PBMC: Peripheral blood mononuclear cell; RA: Rheumatoid arthritis; RANKL: Receptor activator of nuclear factor κB ligand; RF: Rheumatoid factor; SHS: Sharp/van der Heijde score; TNFα: Tumour necrosis factor α.

## Competing interests

WPM is a coinventor of the 14-3-3η technology and Chief Medical Officer of CaRE Arthritis. DvdH received consulting fees and/or research grants from AbbVie, Amgen, AstraZeneca, Augurex Life Sciences Corp, Bristol-Myers Squibb, Centocor Biotech, Chugai Pharmaceutical Co, Daiichi Sankyo, Eli Lilly & Co, GlaxoSmithKline, Janssen Biologics, Merck & Co, Novartis, Novo Nordisk, Otsuka Pharmaceutical, Pfizer, Roche, Sanofi-Aventis, Schering-Plough, UCB and Vertex Pharmaceuticals, and is Director of Imaging ǀ Rheumatology BV. CFA declares no competing interests. RL has been a consultant to or participated on advisory boards for Abbott/AbbVie, Ablynx, Amgen, AstraZeneca, Bristol-Myers Squibb, Centocor Biotech, GlaxoSmithKline, Novartis, Merck & Co, Pfizer, Roche, Schering-Plough, UCB and Wyeth; has received research grants from Abbott, Amgen, Centocor Biotech, Novartis, Pfizer, Roche, Schering-Plough, UCB and Wyeth Pharmaceuticals; has received speaker fees from Abbott, Amgen, Bristol-Myers Squibb, Centocor Biotech, Merck & Co, Pfizer, Roche, Schering-Plough, UCB and Wyeth; and is Director of Rheumatology ǀ Consultancy BV; GB has received research grants and/or speaker honoraria from, and has served on advisory boards of, Abbott Canada, Amgen Canada, Aventis Canada, Bristol-Myers Squibb, Hoffmann-La Roche, Janssen, Novartis Canada, Pfizer, Servier and UCB Canada. PPT became an employee of GlaxoSmithKline after the completion of this study. YG and AM are employees of Augurex Life Sciences Corp. AG is a coinventor of the 14-3-3η technology. RK is a coinventor of the 14-3-3η technology.

## Authors’ contributions

WPM participated in the study design and the drafting and review of the manuscript. DvdH and CFA participated in the study design, provided RA tissue samples, collected and analysed data and participated in the drafting and review of the manuscript. RL participated in the study design, provided RA tissue samples, performed statistical analysis and presentation of the results and participated in the drafting and review of the manuscript. GB provided RA tissue samples as well as clinical and radiographic data, collected and analysed data and reviewed the manuscript. PPT participated in the study design and the drafting and review of the manuscript. YG, AG and RK performed cell signalling and transcriptional studies and reviewed the manuscript. AM participated in the study design, development of the 14-3-3η test, quantification of 14-3-3η levels in patient tissue samples, performed cell signalling and transcriptional studies and participated in the drafting and review of the manuscript. All authors read and approved the final manuscript.

## Authors’ information

PPT is currently with GlaxoSmithKline, Stevenage, UK.
